# INFECTIOUS DISEASE: Americas’ Dengue Escalation Is Real—and Shifting

**DOI:** 10.1289/ehp.118-a117

**Published:** 2010-03

**Authors:** Bob Weinhold

**Affiliations:** **Bob Weinhold**, MA, has covered environmental health issues for numerous outlets since 1996. He is a member of the Society of Environmental Journalists

Although there has been a perceived increase in dengue cases in the Americas since 1980, the epidemiology of this disease has not been well documented. A new analysis of 3 decades’ worth of data indicates, however, that dengue is indeed hyperendemic in the Americas—that is, exhibiting a sustained and growing high incidence. All told, the incidence rate of dengue rose 4.3-fold between the periods 1980–1989 and 2000–2007, the team reports, and the rate of the more severe dengue hemorrhagic fever (DHF) rose 8-fold. Their findings, based on data from the Pan American Health Organization and selected ministries of health, were published in the January 2010 *American Journal of Tropical Medicine and Hygiene*.

Dengue is spread by mosquitoes, predominantly *Aedes aegypti*. It affects an estimated 50 million people annually in about half the world’s countries, and the numbers continue to rise even as many cases go unreported. The 4 known serotypes of the flavivirus that causes dengue induce symptoms such as fever, headache, liver dysfunction, and muscle, joint, or abdominal pain. DHF and dengue shock syndrome, which is frequently fatal, appear to be more likely after a person is reinfected by unique strains of virus of a different serotype.

There is no effective vaccine for dengue or treatment besides rest and drinking plenty of fluids. The disease is believed to have been present in the Americas for more than 200 years and was effectively controlled in the 1950s and 1960s through infrastructural changes such as better public health systems, housing, piped water systems, and systematic pesticide application. Sustained control efforts waned when it appeared the disease had been beaten, and epidemic dengue began a dramatic re-emergence in the late 1970s [see “Dengue Reborn: Widespread Resurgence of a Resilient Vector,” *EHP* 116:A382–AA388 (2008)].

From 1980 through 1989 more than 1 million cases of dengue were reported, mostly in Hispanic Caribbean countries, followed closely by Central America and Mexico. More recently, the number of cases has soared, with more than 4.7 million reported between 2000 and 2007. Nearly two-thirds of these cases occurred in Brazil, Paraguay, Argentina, Chile, and Uruguay; the incidence rate rose more than 15-fold from 1980–1989 to 2000–2007. The rate in the Andean subregion (Bolivia, Colombia, Ecuador, Peru, and Venezuela) increased more than 7-fold. It tripled in the non-Hispanic Caribbean and doubled in Central America and Mexico. The worst incidence of DHF for 2000–2007, at 7.3% of all cases, was in the Andean subregion. For all the Americas, the DHF percentage for 2000–2007 was 2.3%, nearly double the rate for 1980–1989.

There is a consistent connection between the local rainy season and increased cases, but the evidence of a dengue–climate change link remains incomplete. “Global climate has become generally more suitable for dengue over the past fifty years,” says Simon Hales, a senior research fellow at New Zealand’s University of Otago whose areas of expertise include epidemiology and climate variability. “However, observed changes in dengue cannot yet be linked to climate change.”

Duane Gubler, director of Duke University’s Emerging Infectious Disease Research Program in Singapore, says the increased frequency of dengue is largely driven by uncontrolled urbanization and lack of effective vector control in tropical and subtropical developing countries, combined with travel to and from those areas by hundreds of millions of people via modern transportation. Other suspected contributors include increased spread of the 4 serotypes and improved reporting. To help counter the dengue threat, “a vaccine will be an important tool for prevention and control,” says Gubler. “But we should not ignore vector control, especially in view of the emergence of other *Aedes aegypti*–transmitted diseases such as yellow fever and chikungunya.”

